# Mitochondria Dysfunction and Neuroinflammation in Neurodegeneration: Who Comes First?

**DOI:** 10.3390/antiox13020240

**Published:** 2024-02-16

**Authors:** Caterina Peggion, Tito Calì, Marisa Brini

**Affiliations:** 1 Department of Biology, University of Padova, 35131 Padova, Italy; caterina.peggion@unipd.it; 2 Department of Biomedical Sciences, University of Padova, 35131 Padova, Italy; tito.cali@unipd.it; 3 Department of Pharmaceutical and Pharmacological Sciences, University of Padova, 35131 Padova, Italy

**Keywords:** inflammasome, neurodegeneration, Alzheimer’s disease, Parkinson’s disease, amyotrophic lateral sclerosis, mitochondrial dysfunction, oxidative stress

## Abstract

Neurodegenerative diseases (NDs) encompass an assorted array of disorders such as Alzheimer’s disease, Parkinson’s disease, and amyotrophic lateral sclerosis, each characterised by distinct clinical manifestations and underlying pathological mechanisms. While some cases have a genetic basis, many NDs occur sporadically. Despite their differences, these diseases commonly feature chronic neuroinflammation as a hallmark. Consensus has recently been reached on the possibility that mitochondria dysfunction and protein aggregation can mutually contribute to the activation of neuroinflammatory response and thus to the onset and progression of these disorders. In the present review, we discuss the contribution of mitochondria dysfunction and neuroinflammation to the aetiology and progression of NDs, highlighting the possibility that new potential therapeutic targets can be identified to tackle neurodegenerative processes and alleviate the progression of these pathologies.

## 1. Introduction

The significant increase in human life expectancy is certainly an encouraging outcome of medical science advancements; however, it has inevitably augmented the incidence of many age-related disorders, including neurodegenerative diseases (NDs). Worldwide, NDs impact millions of individuals and are recognised as one of the major healthcare problems that will continue to increase in importance due to the general demographic composition of the population and the absence of effective cures [1,2,3]. NDs are typically defined as adult-onset disorders, characterised by the progressive degeneration of neuronal cells in selected areas of the nervous system which determines specific clinical presentation and course, such as cognitive decline, motor impairment, or other neurological symptoms, and eventually death [4,5,6].

NDs are largely sporadic and influenced by a combination of genetic, epigenetic, and environmental factors. However, now we know that NDs characterised by early onset have prevalently a genetic origin, and the mechanisms of their pathogenesis are widely investigated to model the development of sporadic NDs. 

Although of different origin and clinical features, NDs share common altered pathways, including defective protein homeostasis and mitochondria dysfunction. Defective protein degradation by the proteasome system and enhanced protein aggregation are both responsible for the accumulation of insoluble deposits and inclusion bodies in different areas of the brain [7,8,9]. Mitochondria impairment results in bioenergetics defects [10], enhanced formation of free radicals/reactive oxygen species (ROS) and consequent oxidative stress, ER stress, neuroinflammation, defective Ca^2+^ homeostasis [11,12,13,14,15,16], excitotoxicity, and disruption of axonal transport [17].

Growing evidence suggests that, among such altered processes, dysfunctional mitochondria may represent a primary cause, and not merely a consequence, of the neurodegenerative process [18]; therefore, many studies, both in patients and animal models, have investigated the structural and functional abnormalities of these organelles that precede the onset of the histopathological hallmarks of NDs [19]. Thanks to these studies, a link between mitochondrial dysfunction, consequent oxidative stress, and inflammation has been strongly established also in the central nervous system (CNS), where inflammatory response represents a physiological protective process not only necessary to respond to injury or infections but also to counteract the pathogenesis of neurodegenerative processes. However, the robust and long-lasting activation of neuroinflammation has been shown to enhance neurodegeneration.

It is well recognised that the activation of cytosolic protein complexes, called inflammasomes, among which the most characterised is the nucleotide oligomerisation domain (NOD)-like receptor protein 3 inflammasome (NLRP3), leads to local or systemic inflammation [20]. In the CNS, the primary immune sentinels are the microglia which, together with astrocytes and oligodendrocytes, have a fundamental role in supporting inflammatory response [21]. Generally, the activation of microglia occurs as a consequence of a trauma, infection, or any change in neuronal activities and is triggered by the binding of pathogen-associated molecular patterns (PAMPs) or damage-associated molecular patterns (DAMPs) to cell surface receptors (PRRs) [22], which drives the transcription and activation of the inflammasome, leading to the production of inflammatory cytokines, ROS, NO, and eventually cell death through a process called pyroptosis. Uncontrolled and prolonged inflammation could act as a trigger for neuronal damage; thus, a finely tuned equilibrium between anti-inflammatory and proinflammatory activities is essential. 

In this review, we address the intricate relationship between mitochondrial dysfunction, oxidative stress, NLRP3 overactivation, and neuroinflammation in the pathogenesis and progression of Alzheimer’s disease (AD), Parkinson’s disease (PD), and amyotrophic lateral sclerosis (ALS). We additionally discuss recent pieces of evidence showing how targeting mitochondria homeostasis or inflammasome activation could be an effective option for the treatment of such NDs.

## 2. Mitochondria Function and Dysfunction in Health and Neurodegeneration

Mitochondria are plastic and dynamic double-membrane organelles able to adapt to cell metabolic demands (and eventually stress) by varying their size, shape, and number [23]. Because of their endosymbiotic origin, mitochondria share bacterial characteristics such as circular double-stranded mitochondrial DNA (mtDNA) and cardiolipin, a unique phospholipid localised in the mitochondrial inner membrane [24]. Their well-recognised role is to sustain cell bioenergetics: they are defined as the “powerhouses” of the cell, and besides their participation in key metabolic processes, including the metabolism of carbohydrates, fats, and amino acids, they are the major site that produces ATP through the electron transport chain (ETC) and oxidative phosphorylation coupling. Furthermore, mitochondria preserve cell health by exerting a critical function in stress responses and homeostasis maintenance by regulating intracellular calcium homeostasis, managing ROS production, which results as a by-product of the electron leakage of ETC during normal respiration, preventing oxidative stress, and playing a key role in apoptotic activation [23]. Mitochondria do not operate independently but interact with all other organelles by exchanging signals, metabolites, lipids, and calcium, thus executing cellular hub function [25,26,27]. Nonetheless, mitochondria form a dynamic and interconnected structure that spreads throughout the cytoplasm of the cell and results from a continuous process of mitochondrial fusion and fission that allows them to respond to changes in cellular energy demand, adapt to stress, and ensure the proper distribution of healthy mitochondria to daughter cells during cell division. The fusion and fission processes are governed by the interplay between dynamin-related protein 1 (Drp1) and fission 1 (Fis1), which promote fission, and Mfn1, Mfn2, and Optic atrophy 1 (Opa1), that promote fusion [reviewed in [28].

Mitochondria health and proper function are guaranteed by a balance between mitochondrial biogenesis and mitophagy, a process through which damaged or dysfunctional mitochondria (that may produce excessive ROS) are selectively removed from the cell. It involves the engulfment of targeted mitochondria by double-membrane structures called autophagosomes, followed by fusion with lysosomes for degradation. Two key proteins are crucial for mitochondrial quality control machinery: mitochondrial PTEN-induced putative kinase 1 (PINK1) and cytosolic ubiquitin E3 ligase Parkin, and intriguingly, mutations of both of them are linked to familial forms of Parkinson’s disease [29,30]. The accumulation of PINK1 on dysfunctional/depolarised mitochondria allows for the phosphorylation of ubiquitin, which in turn leads to mitochondrial recruitment and the activation of Parkin to ubiquitinate proteins on the outer mitochondrial membrane of damaged mitochondria that are recognised by autophagy receptors [31,32]. On the other hand, mitochondrial biogenesis is crucial for adapting cellular energy conversion to the changing metabolic needs of the cell. It occurs in response to increased energy demands, exercise, or during recovery from cellular stress. Nuclear and mitochondrial genomes coordinate to ensure the synthesis of new mitochondrial proteins. Key regulators of mitochondrial biogenesis include transcription factors such as PGC-1α (peroxisome proliferator-activated receptor gamma coactivator 1-alpha) and NRF1/2 (nuclear respiratory factors), which activate the expression of nuclear-encoded mitochondrial proteins [33]. 

**Figure 1:**
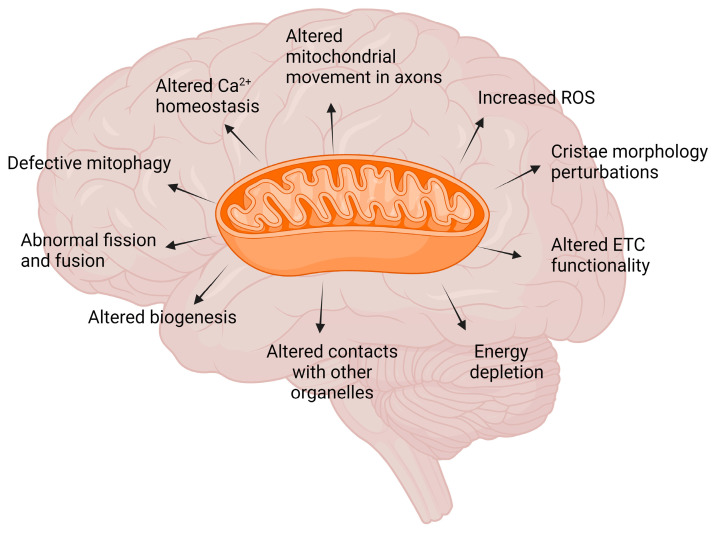
Mitochondrial dysfunction in neurodegenerative disorders. Mitochondria play a central role in cellular metabolism and function and are highly dynamic organelles that respond to environmental stimuli, activating fission, fusion, and mitophagy process. This is particularly important for high-energy-demanding cells as neurons. Several mitochondria abnormalities are characterised by a loss of efficiency in the electron transport chain (ETC) and dysfunction in ATP synthesis machinery, leading to energy depletion and impaired mitochondrial movement in axons. Reactive oxygen species (ROS) are a consequence of the electron transport process, produced as a by-product of oxidative phosphorylation. Mitochondria dysfunction is also linked to defective communication with other cellular organelles and consequent altered Ca^2+^ homeostasis, which could further exacerbate mitochondria damage. Mitochondria dysfunction has been documented in cellular and animal models for NDs as early sign, before the appearance of neurodegenerative phenotype; however, altered mitochondria morphology, cristae organisation, and mitochondria distribution have also been found in postmortem brain of patients. Created with BioRender.com.

Defective mitochondria biogenesis and mitophagy, as well as defective dynamics, are commonly observed in ND models, even before the pathological manifestation of the hallmarks, suggesting that mitochondrial abnormalities not only contribute to their progression but could have an impact on their onset [33,34]. For a general overview, see Figure 1.

### 2.1. Mitochondria Dysfunction in AD

Oxidative stress [35], lipid peroxidation, increased mtDNA modification or deletion, and disruption of Ca^2+^ homeostasis were repeatedly observed both in transgenic (tg) AD model mice and in the brains of AD patients [36,37,38] before the appearance of major AD neuropathological hallmarks, i.e., the deposition of the amyloid-β (Aβ) peptide into amyloid plaques and the phosphorylated tau protein in neurofibrillary tangles (NFTs) [39]. Moreover, reduced mitochondrial membrane potential, ATP synthesis, defects in complex I activity, and impaired respiration have been frequently associated with the early stage of AD [40,41], suggesting that mitochondria dysfunction may be one important trigger for the disease [42,43]. Interestingly, in different AD models, it has been shown that the transcription of the genes encoding amyloid-β precursor protein (APP) and β-Site APP cleaving enzyme 1 (BACE1), that play a key role in the deposition and aggregation of extracellular Aβ, is upregulated by the activation of ROS-sensitive HSF-1 and NF-kb transcription factors or by the activity of stress-activated protein kinase. These findings established a direct link between increased ROS levels and the accumulation of Aβ deposits. Moreover, indirectly, lipid peroxidation caused by oxidative stress has an impact on the activity of Aβ-degrading proteases and Aβ-generating γ-secretase enzyme [44,45]. Data obtained in M17 neuroblastoma cells and in primary rat cortical neuronal cultures demonstrated enhanced tau phosphorylation upon ROS exposure, while antioxidant molecules inhibit it [46,47]. However, the relationship between oxidative stress and tau phosphorylation is still debated. In addition to defects in ETC activity, altered mitochondrial fission and fusion processes and the defective expression of related proteins were reported in postmortem AD brains and fibroblasts from sporadic AD patients [48,49,50]. Interestingly, the enhanced activation of Parkin-mediated mitophagy was observed in the brains of familiar and sporadic AD patients and in cellular and animal AD models [51]. Accordingly, mitochondrial stress response was activated in tissues from individuals with mild and moderate AD. Taking advantage of this observation, Sorrentino et al. demonstrated the promotion of mitochondrial proteostasis by pharmacologically and genetically targeting mitochondrial translation and mitophagy led to a reduction in Aβ aggregation in cell and animal AD models, enhanced survival in a *C. elegans* model, and improved memory function in AD mice [52]. 

It is also interesting to note that, although AD familial cases (which are less than 1% of AD total cases [53]) are principally caused by mutations in genes coding for the amyloid precursor protein (APP) and for the catalytic components of γ-secretase presenilin 1 and 2 (PSEN1 and PSEN2), i.e., the cellular proteases which process APP in Aβ peptide [54], genome-wide association studies (GWASs) have identified several risk loci in genes related to mitochondria functionality. Among them, the gene encoding the pentatricopeptide repeat-containing protein 1, a protein involved in the proper assembly of the mitochondrial ribosome and hence in the translation of mt-DNA-derived mRNAs, and that encoding the TOMM 40 translocator of the outer mitochondrial membrane involved in mitochondrial protein import [55,56,57], are the most relevant. Moreover, an increased expression of genes related to mitochondrial metabolism, morphology, and apoptosis was found both in tg AD mice and in tissue brains derived from AD patients, suggesting that multiple aspects of mitochondrial function are affected in AD [58,59]. 

Taking into consideration all the abovementioned mitochondrial dysfunctions in AD, in addition to the most popular amyloid hypothesis for AD pathogenesis, some authors have proposed the mitochondrial hypothesis [19,60,61]. However, it is still debated whether mitochondrial abnormalities represent a primary cause of AD or if they are rather a secondary consequence of amyloidogenic pathology [19,62,63]. Various studies have suggested that amyloid and tau deposition may be the consequence of mitochondrial dysfunction, as well as that Aβ deposition is responsible for the impairment of mitochondrial transport and fission/fusion processes. In this respect, Aβ has also been recently found to be imported into mitochondria, where it is processed and degraded by the presequence proteases that thus reduce its toxic effect. Interestingly, this proteolytic activity was reduced in mitochondria from AD mice and AD brains [64], possibly as a consequence of increased ROS production and reinforcing the link between oxidative stress and amyloid processing and their synergistic effect [65]. 

### 2.2. Mitochondria Dysfunction in PD

Defective mitochondria function and increased oxidative stress are widely accepted as having a key role also in PD pathogenesis, although the underlying mechanism is still debated [66,67,68,69,70,71].

PD is the second most common progressive ND after AD, and it is characterised by the loss of dopaminergic neurons in the substantia nigra pars compacta (DA SNc) and the accumulation of α-synuclein intracellular aggregates, forming Lewy bodies and Lewy neurites [72,73], which result in both in motor and nonmotor symptoms. 

As AD, PD is a multifactorial ND whose origin is prevalently sporadic, but familial forms of PD account for 10–15% of total cases, and among them, 5% have a Mendelian inheritance. Up to 23 genes have been identified as either causative or representing risk factors for PD. Autosomal dominant PD inheritance is linked to mutations in genes encoding α-synuclein (*SNCA* or *Park1/4),* leucin-rich-repeat kinase 2, LRRK2 *(Park8)*, and VPS35 retromer complex component *(Park17)*, while autosomal recessive PD forms are due to mutations in Parkin *(Park2)*, PTEN-induced kinase 1, PINK1 *(Park6),* and DJ-1 (*Park7)* genes. Moreover, mutations in *GBA1*, the gene encoding the lysosomal β-glucocerebrosidase, causing Gaucher’s disorder, represent the most important risk factors for PD [29,30,74,75,76,77,78,79,80].

The first correlation between PD aetiology and mitochondrial dysfunction was established in the 1980s after the observation that drug users who consumed a synthetic form of heroin, contaminated with the mitochondrial ETC inhibitor 1-methyl-4-phenyl-1,2,3,6-tetrahydropyridine (MPTP), developed symptoms like Parkinson’s disease [81,82,83,84]. Since its original discovery, MPTP-induced toxicity has been repeatedly demonstrated and verified as a model for mimicking PD neurodegeneration both in rodents and primates [85]. Furthermore, MPTP studies have shown that mitochondrial complex I inhibition in DA SNc neurons can result in a clinical phenotype that resembles that of idiopathic PD; the inhibition of complex I by paraquat and rotenone or the genetic ablation of the essential core subunit Ndufs2 subunit of complex I in DA neurons are sufficient to produce progressive, L-DOPA-responsive parkinsonism [82]. The PD link with impaired ETC function was further strengthened by the finding that increased mtDNA aberrations/deletions, that have an impact on the expression of mitochondrially encoded subunits of respiratory complexes, were found in the postmortem SNc tissue of PD patients [86,87].

Interestingly, another crucial factor in establishing a strong connection between mitochondrial dysfunction and PD pathogenesis is the fact that loss-of-function mutations causing genetic forms of PD occur in genes encoding proteins like PINK1, Parkin, and DJ-1, whose function is directly involved in the processes of mitochondrial quality control and oxidative stress protection [88]. PD-related mutations of parkin and PINK1, which are the most frequent cause of autosomal recessive early-onset PD, have an impact not only on the mitophagy pathway but also on mitochondrial biogenesis and fission process. ETC impairment, reduced ATP production, and increased ROS production were observed in fibroblasts derived from patients harbouring PINK1 and Parkin mutations, and mice lacking the two proteins were shown to exhibit defects in complex I activity, reduced Ca^2+^ buffering capacity, and impairments in mitochondrial membrane potential [71]. PD-related mutations in the gene coding for DJ-1, a multifunctional protein mainly involved in counteracting oxidative stress, were shown to lead to elevated mitochondrial oxidant stress and lysosomal dysfunction [89,90]. Abnormalities in mitochondrial shape, dynamics, and high ROS levels, were shown in either lymphoblast derived from DJ-1-PD patients or in mice DJ-1-deficient primary cortical neurons and embryonic fibroblasts [91]. Mutations in the LRRK2 gene, the most prevalent cause of late-onset autosomal dominant PD, were also shown to have an impact on mitochondria function, resulting in an increase in ROS production, alteration in fission and fusion process, reduced mitochondrial membrane potential and ATP production, and increased mitophagy in different PD models (reviewed in [92]). Moreover, it has been found that cortical neurons and familial PD patient fibroblasts with pathogenic LRRK2 mutants display increased mitochondrial calcium uptake and increased mitochondrial calcium uniporter (MCU) levels [93], which may contribute to their susceptibility to mitochondrial calcium overload and damage. Finally, also, α-synuclein has mitochondria as a target. Many studies are available in the literature that show its role in physiological and pathological conditions by interfering with Ca^2+^ homeostasis, mitochondrial protein import, and many other mitochondria-related activities [94,95,96]. Very recently, the Surmeir group monitored mitochondria function in DA SNc after the stereotaxic injection of α-synuclein preformed fibrils into mice brains and found that in addition to inducing neuronal loss, α-synuclein injection decreased mitochondrial gene expression and increased oxidant stress and bioenergetic defects, thus impacting on the autonomous spiking of dopaminergic neurons [97].

### 2.3. Mitochondria Dysfunction in ALS

ALS is a progressive ND that principally targets upper and lower motor neurons (MNs) in the motor cortex, brainstem, and spinal cord, causing muscle wasting, atrophy, and respiratory failure [98,99]. A pathological hallmark is the presence of cytoplasmic inclusions whose major components are fused in sarcoma/translocated in liposarcoma RNA-binding protein (FUS/TLS), TAR DNA-binding protein 43 (TDP-43), and the translational product of intronic repeats in gene C9ORF72. Like AD and PD, ALS cases are prevalently sporadic, but 10% of them are familial with autosomal dominant transmission. Mutations in the genes encoding the abovementioned proteins and in that encoding the Cu/Zn superoxidase dismutase 1 (SOD1) enzyme were linked to most of the inherited forms of ALS, and their identification has allowed for ALS disease models to be generated and pathological mechanisms to be unveiled. In addition to these causative mutations, polymorphisms in up to 17 genes were definitively recognised to be associated with an increased risk of ALS. Intriguingly, some of the products of these genes are involved in processes like neuroinflammation and oxidative stress response (https://alsod.ac.uk/ accessed on 9 December 2023). As for other NDs, the aetiology of ALS is multifactorial, and among other deregulated pathways, mitochondria dysfunction has been reported to occur as one of the earliest pathophysiological events. Evidence for mitochondrial damage and oxidative stress was first unveiled in a mouse model of ALS overexpressing the mutant G93A-SOD1 [100,101], although similar findings were then found also in other non-SOD1 ALS models (reviewed in [102,103]). Since SOD1 is a ubiquitous superoxide scavenging enzyme, the obvious correlation between the presence of mutations and increased oxidative stress was the possibility that its enzymatic activity could be compromised. Surprisingly enough, most of the over 180 identified point mutations (for a list, see https://alsod.ac.uk/output/gene.php/SOD1, accessed on 15 January 2024) have been shown to affect protein stability and folding, making SOD1 more prone to aggregation. In the case of most mutations, including common G93A and G37R, the pathology develops in the presence of a fully active enzyme, thus suggesting that ALS-related SOD1 mutations lead to a toxic gain of function by promoting aggregates formation within the cells, but that they also contribute to pathology with a loss-of-function mechanism by reducing the amount of functional enzyme and thus promoting oxidative stress [104,105]. Hence, the exact mechanism through which SOD1 mutations induce MN degeneration is still debated: gain or loss-of-function, or both. In addition, mutant SOD1 was found to accumulate within the mitochondria, contributing to their functional and structural alteration, which further compromises the ability of the cells to counteract oxidative stress [103].

Changes in mitochondria morphology are one of the first hallmarks observed in motor neurons of ALS patients [106] and in cell or animal TDP-43 or SOD1-related models of ALS [107,108,109], together with the reduction in cellular respiration and ATP production, due to deficits in complex I, II, III, and IV activity that have been found in different models, i.e., the postmortem spinal cord of sporadic ALS patients or from skeletal muscle and lymphocytes derived from them [110]. Increased levels of ROS and ROS-induced damage to DNA, RNA, proteins and lipids [111], and perturbations of calcium homeostasis have been observed in in vitro and in vivo ALS models harbouring mutations in SOD1, TDP-43, and FUS/TLS and in the motor nerve terminals of ALS patients [112,113,114,115,116,117,118]. Furthermore, a number of in vitro and in vivo experimental models have reported the impaired anterograde and retrograde transport of mitochondria, which leads to the aberrant clustering of mitochondria along the axon [119]. Similarly, such mislocalisation was observed in spinal cord sections from ALS patients [106]. However, the mechanisms underlying such alterations are not completely understood and could be due to defects in the axonal transport machinery caused by the destabilisation of the microtubules, protein aggregation, and/or energetic defects due to mitochondrial damage [120]. A reduced expression level of Miro1, a key regulator of mitochondrial movement, was found both in the spinal cord tissue of ALS patients and in different ALS Tg mice models [121]. An increased level of DRP1 and Fis1 and a concurrent decreased level of Opa1 were observed both in cell lines overexpressing the ALS-related G93A-SOD1 protein and in an ALS mouse model, thus suggesting changes also in fusion and fission processes [122].

## 3. Neuroinflammation and Inflammasome Activation through Mitochondria Dysfunction

The characterisation of mitochondria dysfunction in different NDs has represented an intense field of research in recent years, and the finding that mitochondria damage plays a direct role in neuroinflammation processes opens up new perspective for therapeutical interventions to counteract neurodegenerative processes. In the past, neuroinflammation was essentially considered to occur because of other pathologies and to exacerbate neuronal damage; now it is widely considered to causally contribute to neurodegeneration. Under physiological conditions, neuroinflammation is a protective response to insults of different origin that activate CNS-resident glial cells, i.e., mainly microglia, which represent the innate immune cells of the nervous system [21]. While, if managed, it represents a defence mechanism that protects the brain by removing or inhibiting pathogens, when it becomes chronic or sustained, it fails to resolve and turn into a key driver of the neurodegenerative process, leading to neuronal death. Microglia are widely dispersed throughout the brain in a resting state. Pathogenic stimuli like protein aggregates or neuronal death, but also genetic and environmental factors, induce the activation of microglia, leading to morphological changes, the secretion of proinflammatory cytokines and ROS, and the adoption of a phagocytic trait [123]. Activated microglia can polarise in various phenotypes depending on the signals received, ranging from opposite proinflammatory (M1) and anti-inflammatory (M2) phenotypes. During the M1 phase, microglia release proinflammatory mediators, including TNF-α, IL-1β, IL-6, nitric oxide (NO), ROS, chemokines (such as macrophage inflammatory protein 1α, monocyte chemoattractant protein-1 (MCP-1)), and neurotoxins [124]. When such a robust proinflammatory response endures over an extended period (e.g., in chronic illnesses like neurodegenerative disorders), it may exacerbate neuronal damage [124]. Conversely, the M2 response elicits anti-inflammatory and reparative responses through the release of anti-inflammatory factors (e.g., IL-4, IL-10, transforming growth factor-β (TGF-β), insulin-like growth factor 1, and brain-derived neurotrophic factor), protecting against brain damage. A proper balance between these two phenotypes is crucial for the resolution of an inflammatory state, while its impairment can lead to severe pathological outcomes.

**Figure 2:**
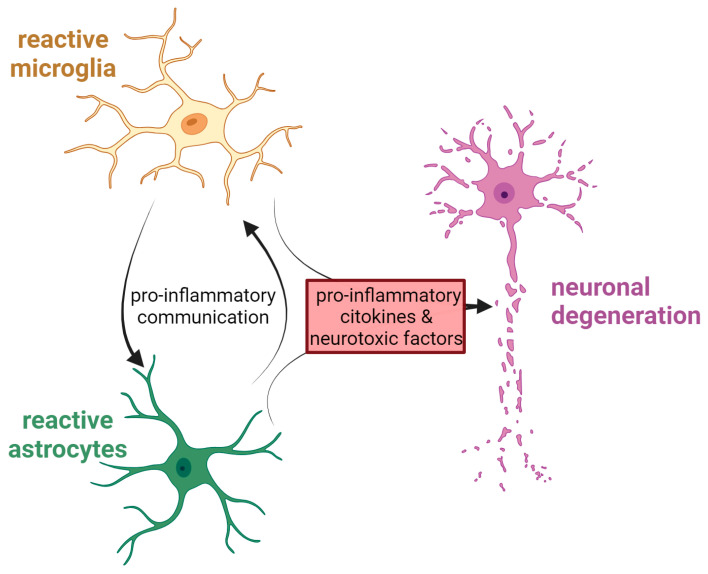
Interplay among CNS cells during neurodegeneration. The cartoon shows the harmful relationship that occurs in neurological disorders between activated astrocytes, microglia, and neurons. Created with BioRender.com.

Another cell population deeply involved in the neuroinflammatory response is that of astrocytes. Changes in their shape, abundance and activity result from the process known as astrocytosis, which is commonly observed in many NDs [125]. As microglia, astrocytes can exist in a different state and release a variety of inflammatory modulators, such as cytokines or neurotrophic factors, that can either be neurotoxic or neuroprotective [126]. Astrocytes participation in the progression of NDs could be due to a combination of their loss of homeostatic function at the synapse and their gain of function, i.e., the acquisition of a reactive phenotype (astrogliosis) in response to the accumulation of protein aggregates inside the cells, which is often observed in parallel to loss of synapses. During pathogenesis, moreover, the cross-talk between astrocytes and microglia seems to be particularly important, and it occurs possibly through different mechanisms, such as direct cell-to-cell contact, cytokines, chemokines, neurotrophic factors, nanovesicles, and non-vesicular-mediated secretion [127]. Figure 2 summarises the deadly cross-talk between glial cells and neurons during neurodegeneration.

In microglia, damage to mitochondria represents a source of inflammation through the activation of high-molecular-weight cytosolic complexes belonging to nonspecific immunity known as inflammasomes that assemble and become a platform, leading to the amplification of inflammatory cascade [20]. Inflammasomes are usually composed of three actors, (i) the sensor, i.e., proteins belonging to the nucleotide binding oligomerisation domain (NOD)-like receptor (NLR), (ii) the bipartite adapter apoptosis-associated speck-like protein (ASC) that contains a caspase recruitment domain (CARD), and (iii) the effector protease caspase-1 [20,128]. NLRP3 inflammasome is the most characterised, and its activation requires two distinct and parallel phases: priming and activation/oligomerisation. During priming, the recognition of PAMPs, DAMPs, or endogenous cytokines by Toll-like receptors (TLRs) and cytokine receptors (e.g., tumour necrosis factor receptor (TNFR)) induces translocation into the nucleus of the nuclear factor kappa B (NF-κB) and the activation of the transcription of both inactive NLRP3 and pro-IL-1β. After that, different DAMPs (e.g., Ca^2+^ influx, K^+^ efflux, protein aggregates, oxidised mtDNA, ATP, reactive oxygen species (ROS), and lysosomal rupture [128,129,130]) cause the assembling of NLRP3 complex into NLRP3-ASC oligomers which form a higher-order structure called “speck” that recruits the inactive pro-caspase-1 [20]. Once the protease is activated, it can cleave proinflammatory IL-1β and IL-18 and the gasdermin D protein (GSDMD), whose N-terminal domain translocates into the plasma membrane, oligomerises, and forms pores that allow for both the release of proinflammatory cytokines and the initiation of pyroptotic cell death [20,129,130]. Cytosolic mtDNA is the most well-known mitochondrial DAMP. When it is released upon mitochondria damage, it activates cyclic GMP-AMP synthase (cGAS), the primary cytoplasmic DNA sensor, which synthetises cyclic GMP-AMP (cGAMP). cGAMP binds to the Stimulator of Interferon Gene (STING) protein, which activates tank-binding kinase 1, and in turn promotes the oligomerisation of NLRP3 and the expression of several interferon genes [131]. mtROS are also important DAMPs, although their exact mechanism for inflammasome activation is not fully understood [132]. Evidence suggests that they can cause the dissociation of thioredoxin (TRX) from TRX interaction protein, leading to its binding to the leucine-rich repeat region of NLRP3, with consequent NLRP3 inflammasome activation [133]. Another potential mechanism involves an increase in mtDNA mutations due to augmented mtROS levels, which leads to the activation of the cGAS-STING pathway [134]. Also, ATP, through its binding to the purinergic receptor P2X7, which causes Na^+^ and Ca^2+^ entry and a consequent decrease in intracellular K^+^, induces the activation/oligomerisation of NLRP3 [20]. Additionally, it has been shown that free molecules of cardiolipin, i.e., the major lipid of the mitochondrial inner membrane [135] can activate NLRP3 and inflammation [136], and that extracellular cardiolipin stimulates phagocytosis by microglia [137].

All these elements strongly underline the link between mitochondria damage and neuroinflammation, which is a vicious circle; inflammation caused by mitochondria dysfunction is responsible for the continuous production of toxic mediators, such as ROS and proinflammatory cytokines, that in turn affect mitochondrial activities, thus boosting ROS production, neuroinflammation, and neuronal death [138].

In the following paragraphs, the main causes of NLRP3 inflammasome activation in AD, PD, and ALS are discussed in more detail from the perspective of delineating the possible causative role for genetic or environmental conditions, which are both responsible for mitochondria damage and triggering neuroinflammatory response.

### 3.1. Neuroinflammation and Inflammasome Activation in AD

The first piece of evidence relating to the involvement of microglia in AD came from the analysis of several GWASs, showing that some genetic loci that confer an increased risk of AD are linked to microglia function and/or neuroinflammatory mechanisms [139]. Among these, several variants in the triggering receptor expressed on Myeloid cells 2 (TRM2) were reported. TRM2 is a microglial surface receptor crucial for the local proliferation of microglia near to amyloid deposits [140] and the clearance of Aβ oligomers [141]. Also, polymorphisms in CD33, a type I transmembrane receptor expressed both in microglia and in immune cells in peripheral tissues, were associated with AD risk [142]. Interestingly, upregulation of CD33 expression was found in AD patients’ brains, while the ablation of its gene mitigated Aβ amyloid plaque burden and pathology in AD model mice [143].

The activation of NLRP3 in microglia is widely implicated in AD pathogenesis [144,145]. For instance, elevated levels of NLRP3 were found in the blood, cerebro spinal fluid, and brain tissues of AD patients [146].

**Figure 3:**
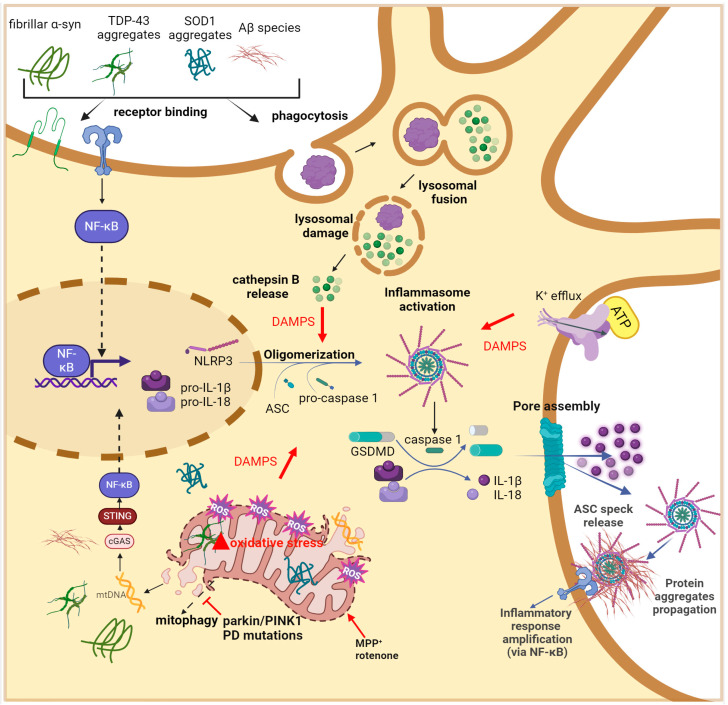
Mechanisms of microglial NLRP3 inflammasome activation in NDs. During the priming signal, distinct protein clumps (Aβ in AD, α-synuclein in PD, SOD1, and TDP-43 in ALS) bind to plasma membrane receptors of microglia, therefore initiating the expression of proinflammatory cytokines (e.g., pro-IL1β and pro-IL-18) and NLRP3 via NF-kB transcription factor activation. The inflammasome activation signal is supplied by lysosomal cathepsin B released into the cytoplasm upon lysosomal damage caused by the phagocytosis of protein aggregates and by DAMPs (ATP, mtROS, cytosolic mtDNA) signalling pathways that promote NLRP3 assembly and caspase-1 activation. Consequently, proinflammatory IL-1β and IL-18 are secreted through the gasdermin D (GSDMD) pore assembly, amplifying the inflammatory process. ASC specks are also released through GSDMD pore into the extracellular environment, thus acting as a scaffold to cross-seed misfolded protein aggregates that further enhance proinflammatory phenotype. Created with BioRender.com.

NLRP3 activation occurs through several intertwined ways. The most important triggers are Aβ aggregates (i.e., oligomers, protofibrils, and fibrils, but not monomers) [147,148] and tau neurofibrillary tangles [149]. Nonetheless, the entire process of neuroinflammation is amplified by the leaking of mitochondrial components brought on by pathology-related mitochondrial malfunction. The Aβ oligomers-mediated activation of NLRP3 can occur in different ways. One regards their recognition and binding to microglial surface receptors (CD36, TLR4, and TLR6), thus inducing Nf-kB activation and the consequent transcription of NLRP3 and proinflammatory interleukin-coding genes. [150,151,152]. Another relies on phagocytised Aβ that leads to cathepsin B release by disrupted lysosomes, which in turn acts as DAMPs for the activation of NLRP3 inflammasome [153]. Furthermore, Aβ-induced neuronal cell death causes the release of ATP that, through binding to the P2x7 receptor in microglial cells, causes the efflux of K^+^ and consequent NLRP3 activation [154,155]. A major consequence of AD neuroinflammation is that Aβ-induced microglial NLRP3 inflammasome activation results in the extracellular release of ASC specks, which may act as binding cores for Aβ, promoting the formation and propagation of Aβ oligomers and aggregates and providing evidence in favour of the theory that AD pathology worsens through inflammasome activation [156,157]. See Figure 3.

Interestingly, the deficiency of NLRP3 in APP/PS1 AD Tg mice was demonstrated to ameliorate spatial memory loss and reduce hippocampal and cortical Aβ levels and plaque formation [157]. Similarly, both the depletion of ASC protein and treatment with the MCC950 inhibitors of NLRP3 in Tau Tg mice (carrying the AD-related mutation P301S in the Tau protein) were shown to reduce tau pathology [158].

### 3.2. Neuroinflammation and Inflammasome Activation in PD

Both transgenic α-synuclein PD models and several toxin-based PD models exhibit significant microgliosis [159]. However, although microglial activation is believed to play a major role in PD onset and progression, a direct correlation between DA neuron loss and microglial activation has not been demonstrated. High expression of NLRP3 and extensive inflammasome activation were reported both in postmortem brains of PD patients [160,161,162] and in animal models of PD [163,164].

Different pathways of inflammasome activation have been reported to occur in microglial cells during PD progression. The pathogenic misfolded α-synuclein, which accumulates and spreads over the PD disease course, is the most prevalent trigger of NLRP3 inflammasome activation, leading to the overproduction of proinflammatory cytokines. It was demonstrated that neurons release exosomes containing aggregated α-synuclein that are endocytosed and recognised as DAMPs by microglia, with the consequent activation of NLRP3 inflammasome, cytokines production, and release into the extracellular space, thus perpetrating dopaminergic neuron degeneration [165,166]. On the other hand, α-synuclein can bind to TLR2 and TLR5, thus leading to the activation of NF-κB and transcription of NLRP3 and proinflammatory cytokines. Interestingly, the knock-down of NLRP3 or its inhibition was shown to diminish microglial activation as well as α-synuclein pathology and DA degeneration, indicating that anti-inflammatory treatment strategies can be a therapeutic option for PD [167,168]. However, inflammatory response has also been found in the absence of α-synuclein aggregates, thus supporting the possibility that it is not just a consequence of them.

PD animal models generated by the treatment with complex I inhibitors displayed potentiated NLRP3 inflammasome activation, ASC speck formation, and pro-IL-1β processing to IL-1β, thus suggesting that NLRP3 inflammasome signalling could be induced by mitochondria damage and contribute to the dopaminergic neurodegenerative process [169]. A direct link between NLRP3 activation and PD came from the observation that loss-of-function mutations of parkin and Pink1 protein affect physiological NLRP3 ubiquitination and lead to NLRP3 inflammasome assembly [170]. NLRP3 is a natural substrate of parkin: by targeting it for proteasomal degradation, parkin inhibits inflammasome priming. A loss-of-function of parkin is responsible for the abnormal accumulation of NLRP3 with its spontaneous activation. Activation of the NLRP3 inflammasome occurs in parkin-deficient DA neurons also by an additional pathway: the accumulation of the parkin substrate PARIS leads to mitoROS generation and thus the assembly of the NLRP3 inflammasome complex, which results in increased levels of GSDMD cleavage, rather than detectable IL-1β or IL-18 secretion, suggesting that the neurons might be dying via pyroptotic cell death [170]. See Figure 3.

As in AD, the inhibition of NLRP3 inflammasome assembly prevents the degeneration of DA neurons both in familial and sporadic PD models [168]; these results again suggest how intervention on NLRP3 could be important in ameliorating PD onset and progression.

### 3.3. Neuroinflammation and Inflammasome Activation in ALS

Neuroinflammation is uniformly present in end-stage ALS pathology, but imaging studies on patients and rodent models support the idea that neuroinflammation begins early in disease pathogenesis, suggesting its contribution to the progression of the disease [171,172]. A chronic autoinflammatory state characterised by elevated levels of proinflammatory cytokines, such as tumour necrosis factor-alpha (TNF-α) and interleukin-1 beta (IL-1β), and other nuclear factor kB (NF-kB)-related cytokines, have been observed in the CNS of individuals with ALS and animal models of ALS [173,174,175]. This impinges on the blood–spinal barrier, allowing for immune cells and molecules to enter the spinal cord more easily, thus further exacerbating the neuroinflammation process.

The upregulation of NLRP3 inflammasome was widely demonstrated in the microglia of C9orf72- [176], hSOD1-, and hTDP-43-related ALS mouse models [177,178,179], and increased amounts of NLRP3, ASC, and caspase 1 were found both in postmortem tissue and in the serum of ALS patients [180,181]. Transcriptome analysis performed in a C9orf72 ALS mouse model and C9-ALS patients demonstrated the activation of interferon-responsive proinflammatory microglial [182].

Recently, the group of Pasinetti [183] showed that the genetic ablation of NLPR3-mediated innate immunity in the C9orf72 ALS mouse model significantly attenuates cerebral cortex inflammation, mortality, and neurodegeneration, and most notably mitigates behavioural impairments, suggesting that tackling NLRP3 activation may be a promising disease-modifying therapy for ALS.

Interestingly, in addition to canonical NLRP3 activation by mutant TDP-43 via CD14-mediated microglial NF-κB pathways [184], recently, it was demonstrated that TDP-43 cytosolic aggregates cause the activation of the NLPR3 pathway through the release of mtDNA into the cytoplasm, thus activating the cGAS/STING pathway. See Figure 2. Notably, it was shown that the blockage of STING either in vivo or in vitro inhibits inflammation and ALS-related neurodegeneration [185].

## 4. Conclusions

The view that NDs were essentially disorders of protein homeostasis characterised by the presence of aberrant protein accumulation, which induced cell damage, has dramatically changed in recent years. It is now clear that many additional processes concomitantly occur to play the role of initiator factors. Mitochondria dysfunction, oxidative stress, protein misfolding, and chronic inflammatory response are the major strictly interconnected actors, mutually influencing each other during the progression of the disease. Their intricate relationship contributes to the difficulty of finding a resolutive treatment for NDs. Understanding their role and their interplay is challenging but crucial for developing effective treatment options for modifying the direction of neurodegenerative disease. Recently neuroinflammation has been proposed as a main pathogenic pathway in NDs. At the molecular level, neuroinflammation is mainly triggered by redox status. Mitochondria are responsible for both generating ROS and responding to ROS-induced cellular changes. Hence, mitochondrial dysfunction can be both the leading cause of neuroinflammation and can be induced by it. The accumulation of damaged mitochondria can activate NLRP3 inflammasome-dependent inflammation in microglia, and damaged neurons are responsible for releasing DAMPs, such as mtDNA, in the extracellular environment that act as an inflammatory boost, leading to a vicious inflammatory cycle. Several reports, based on experimental cell and animal models for NDs, indicate that the inhibition of NLRP3 inflammasome could be an innovative therapeutic strategy to counteract AD, PD, and ALS. However, anti-inflammatory therapy failed to delay disease progression in clinical trials, pointing out the complex role of inflammatory signalling in neurodegeneration and the necessity of precisely identify timing, cell specificity, and target molecules to reduce the detrimental role of enhanced inflammation while ensuring the beneficial role of inflammatory response.

We choose a provocative title for this review: Mitochondria dysfunction and neuroinflammation in neurodegeneration: who comes first? Unfortunately, we still do not have a clear answer to this question. A more complete understanding of the crosstalk between the underlying cellular and molecular mechanisms of neurodegeneration will be crucial to consider the possibility that interfering with the interactive signalling pathway between mitochondrial dysfunction and neuroinflammation could represent a disease-modifying approach.
